# Supplementary material for: Exact broadband excitation of two-level systems by mapping spins to springs
Source: Nat Commun. 2017 Sep 5;8:446. doi: 10.1038/s41467-017-00441-7 (PMC5585281; doi:10.1038/s41467-017-00441-7)
Supplement: Supplementary file 1 — Supplementary Information [file 41467_2017_441_MOESM1_ESM.pdf]

Description of Supplementary Files

File Name: Supplementary Information
Description: Supplementary Figures, Supplementary Notes and Supplementary References

File Name: Peer Review File

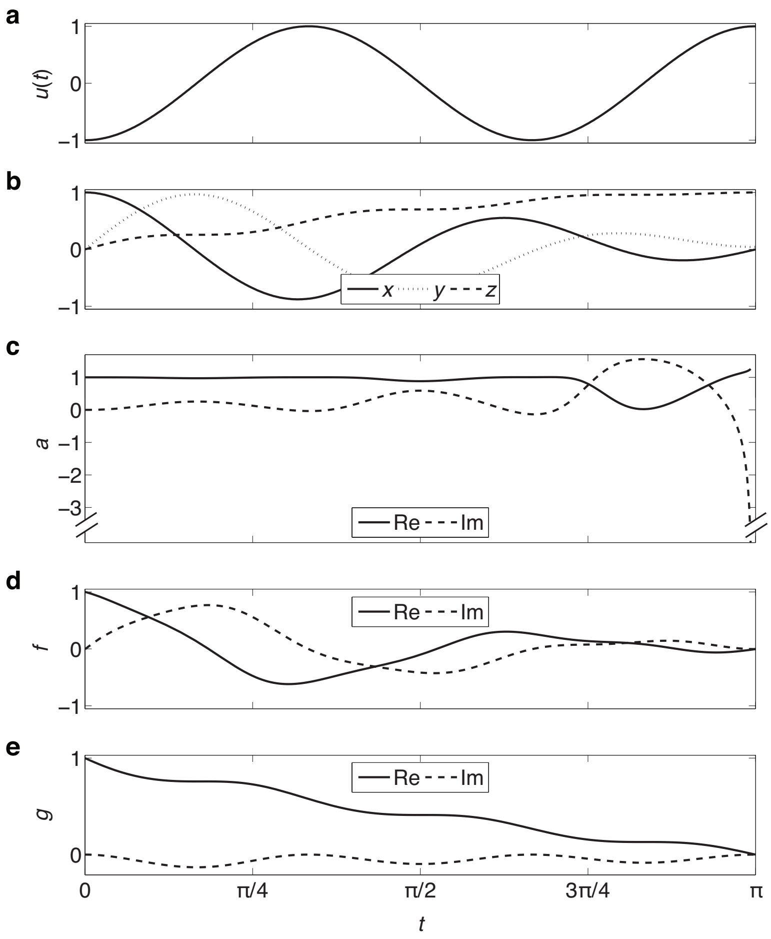

Supplementary Figure 1: **Control of a single spin.** (a) The minimum-energy control $u^*(t) = -\cos(3t)$ as in (9) for $\omega = 3$ and $T = \pi$ and (b) the corresponding spin trajectory $(x(t), y(t), z(t))$ and the evolutions of the complex-valued functions (c) $a(t)$, (d) $f(t)$, and (e) $g(t)$ following $u^*(t)$. Note that the final state of a, $a(T)$, is large since $m(T)$ approaches to 0.

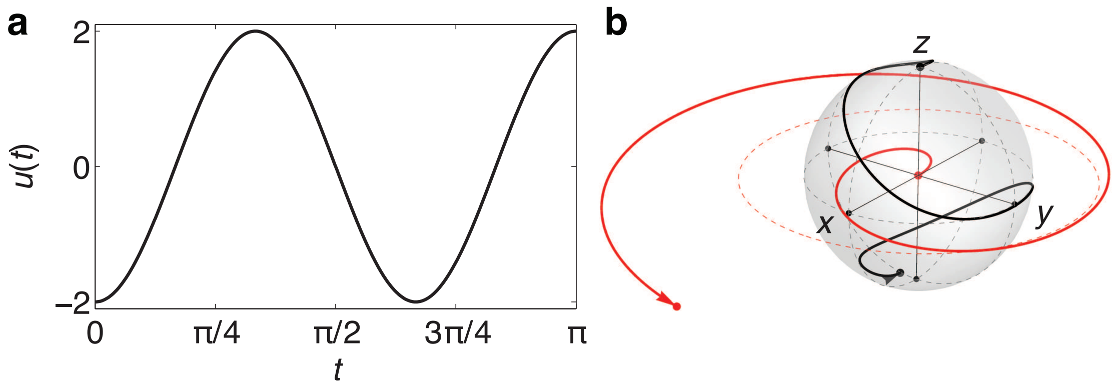

Supplementary Figure 2: **Inversion of a single spin.** (a) The minimum-energy control steering the spring from $(0,0)'$ to $(\pi,0)'$, with (b) the corresponding trajectories of the spring (red) and spin (black) for $\omega = 3$ and $T = \pi$, where the final z magnetization of the spin is -0.9851. The final z magnetization can be made arbitrarily close to -1 by selecting the product ωT appropriately (see Supplementary Note 4); for parameters $\omega = 3$ and $T = 10\pi$, $z(T) = -0.9999$.

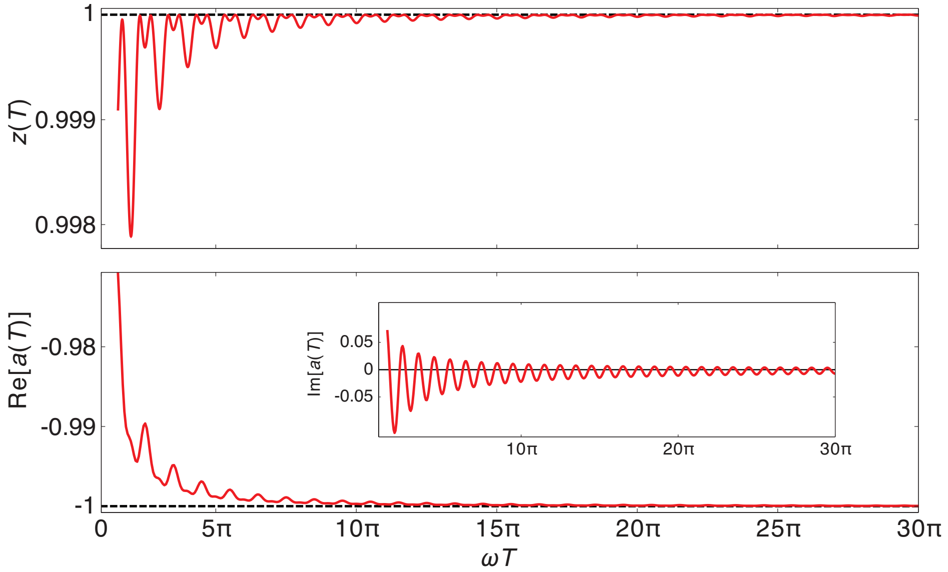

Supplementary Figure 3: **Asymptotic exactness.** The minimum energy control $u^*(t)$ evidences (a) improving final z magnetization as the product ωT increases. An alternative derivation provides insight into why this is by revealing that (b) the asymptotic behavior of $a(T)$ drives this performance improvement. For example, when $\omega T = 30\pi$, $z(T) = 0.99999$.

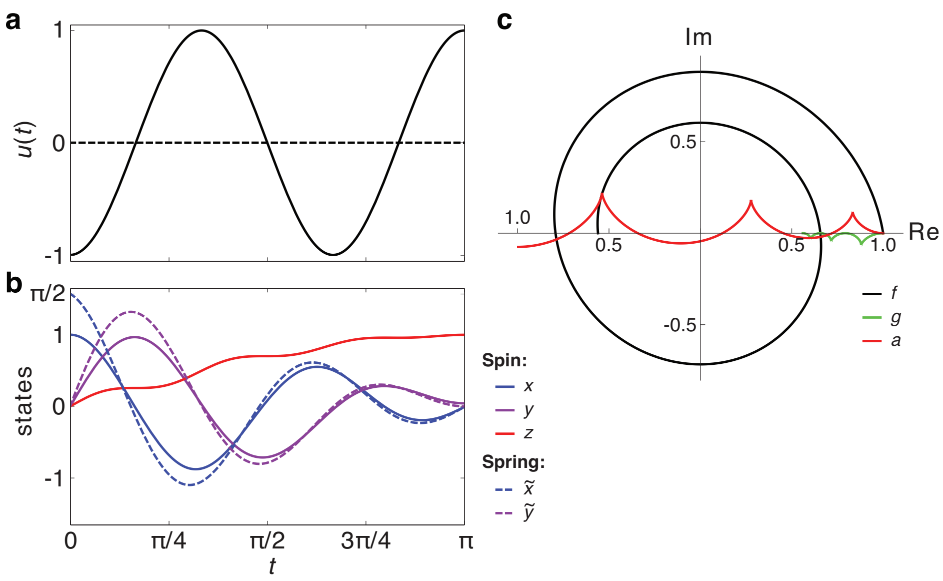

Supplementary Figure 4: **Control of a single spin, revisited.** (a) The minimum energy control $u^*(t) = -\cos(3t)$ as in (9) for $\omega = 3$ and $T = \pi$, (b) the corresponding spin $(m(t), z(t))$ and spring $\mathbf{X}(t)$ trajectories, and (c) the evolutions of the complex-valued functions $f(t)$, $g(t)$, and $a(t)$ following $u^*(t)$. Since the minimum energy control $u^*(t)$ satisfies (6), the final value of g is close to $g(T) \approx \frac{4}{4+\pi} \approx 0.56$. The control also drives $a(T) \approx -1$. The integral condition guarantees the spring is driven to $(0,0)'$ and when $u^*(t)$ additionally satisfies $a(T) \to -1$ the spin is driven to $(0,0,1)'$.

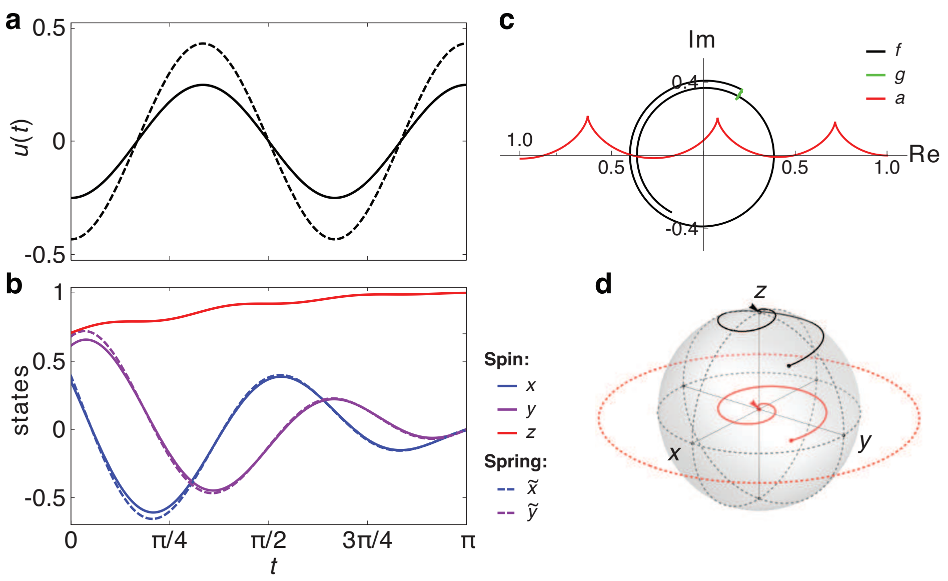

Supplementary Figure 5: **Arbitrary rotation angles.** The combined minimum energy controls $\alpha(t) = u^*(t) + iv^*(t) = -\frac{1}{4}\cos(3t) - i\frac{1}{4}\cos(3t)$ executes a transfer of the spin from spherical coordinates $\theta = \frac{\pi}{3}$ (azimuthal angle), $\phi = \frac{\pi}{4}$ (polar angle), i.e., $\mathbf{M}(0) = \left(\frac{1}{2\sqrt{2}}, \frac{\sqrt{3}}{2\sqrt{2}}, \frac{1}{\sqrt{2}}\right)'$, to $\theta = 0$, $\phi = 0$, i.e., $\mathbf{M}(T) = (0, 0, 1)'$, for $\omega = 3$ and $T = \pi$. These controls are designed separately as described in (79) and (80) to drive the spring from $\tilde{m}(0) = \frac{\pi}{8} + i\,\frac{\pi\sqrt{3}}{8}$ to $\tilde{m}(T) = 0$. We show (a) the control pulse, (b) the corresponding spin $(m(t), z(t))$ and spring $\mathbf{X}(t)$ trajectories, (c) the evolutions of the complex-valued functions $f(t)$, $g(t)$, and $a(t)$ following $\alpha(t)$, and (d) the trajectories (spring in red; spin in black) visualized on the sphere. Notice that the values of $f(0)$ and $g(0)$ are set to accomodate the different initial starting state. The control achieves a transfer with performance $z(t) = 0.99998$. This example, and others like it, empirically demonstrates that the framework presented here is able to generalize to both the setting of arbitrary rotation angles as well as the design of two controls to execute these rotations in arbitrary directions.

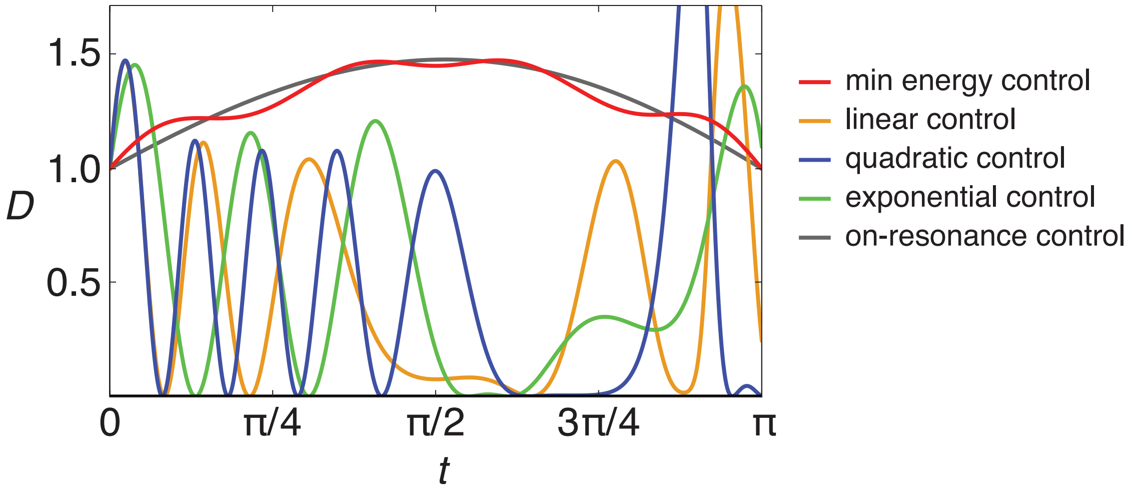

Supplementary Figure 6: **Feasibility of pulse designs.** The trajectory of the discriminant of D as given by (85) resulting from the minimum-energy control; a linear control, $u(t) = \frac{9}{4}t - \frac{9}{8}\pi^2$; a quadratic control $u(t) = \frac{9}{4}t^2 - \frac{9}{8}\pi^2 + \frac{1}{2}$; and an exponential control control $u(t) = \frac{5\pi}{1+e^{\pi}}e^t - \frac{9}{4}$ that steer the spring from $(\pi/2, 0)'$ to $(0, 0)'$. The performance (the z magnetization at $T = \pi$) following each control is 0.9991, -0.7647, 0.2127, -0.4205, 0.9525, respectively (the performance of the exponential control, 0.9525, is a coincidence at these parameter values). Recall (see Supplementary Note 4) that the performance of the minimum energy pulse can be made arbitrarily close to 1 (e.g., increasing the duration to $T = 10\pi$ yields a performance $z(T) = 0.999991$). The well-known on-resonance pulse, $\alpha(t) = -\frac{1}{2}e^{i3t}$, which uses two controls ($u(t) = -\frac{1}{2}\cos 3t$, $v(t) = -\frac{1}{2}\sin 3t$) to perform exact excitation of a single spin (but is not extendable to the broadband case) is also shown as a comparison and also satisfies the discriminant condition in (85).

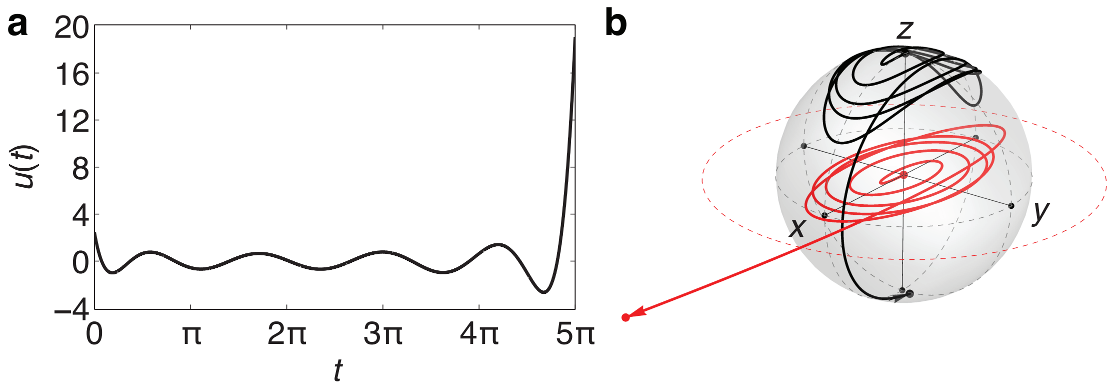

Supplementary Figure 7: **Inversion of a spin ensemble.** The broadband minimum-energy control steers a family of harmonic oscillators with frequencies $-1 \leq \omega \leq 1$ from $(0, 0)'$ to $(\pi, 0)'$, with the corresponding trajectories of the harmonic oscillator (red) and nuclear spin (black) for $\omega = -1$. This pulse achieves an average magnetization inversion, over the frequencies $-1 \leq \omega \leq 1$ of -0.9984.

Supplementary Note 1: Optimal Steering of Springs

The major contribution of this study is to reveal a dynamic connection between the evolution of nuclear *spins* and harmonic oscillators (*springs*) driven by the same external control input. In particular, we are able to create 90° and 180° pulses used commonly in nuclear magnetic resonance (NMR) by designing controls that steer a spring between specific states. The linearity of the spring dynamic enables us to obtain simple analytic expressions for these NMR pulses. We extend this insight to spin and spring ensembles in order to design broadband NMR pulses that compensate for a distribution of spin (and spring) frequencies. Using this technique in conjunction with efficient numerical optimization we can design pulse sequences that also satisfy specific experimental requirements, such as a bound on the radio-frequency (RF) amplitude or the total energy of the pulse.

The well-known simple harmonic oscillator obeys a dynamic described by $\ddot{\tilde{y}} + \omega^2 \tilde{y} = \tilde{u}$, with $\tilde{y}$ the position of the oscillator (or spring) and $\tilde{u}$ is a forcing term. If we let $\tilde{x} = \frac{1}{\omega}\dot{\tilde{y}}$, then $\dot{\tilde{y}} = \omega\tilde{x}$ and $\dot{\tilde{x}} = \frac{1}{\omega}\ddot{\tilde{y}} = -\omega\tilde{y} + u$, where $u = \frac{\tilde{u}}{\omega}$. Therefore, a forced, nondamped harmonic oscillator can be modeled as a linear dynamical system of the form,

$$\frac{d}{dt}\mathbf{X}(t) = \mathbf{A}\mathbf{X}(t) + \mathbf{B}u(t), \tag{1}$$

where $\mathbf{X} = (\tilde{x}(t), \tilde{y}(t))'$ represents the state and $'$ denotes the transpose operation,

$$\mathbf{A} = \begin{bmatrix} 0 & -\omega \\ \omega & 0 \end{bmatrix}, \quad \mathbf{B} = \begin{bmatrix} 1 \\ 0 \end{bmatrix}, \tag{2}$$

in which ω is the frequency of the harmonic oscillator, and $u : [0, T] \to \mathbb{R}$, $T \in (0, \infty)$, is an external input (control), which is (piecewise) continuous on $[0, T]$. We consider steering this harmonic oscillator from the initial state $\mathbf{X}_0 = (\tilde{x}(0), \tilde{y}(0))' = (\frac{\pi}{2}, 0)'$ to the origin, $\mathbf{X}_\mathrm{F} = (0, 0)'$, at a finite time T. Applying the variation of constants formula [1] to (1) yields

$$\mathbf{X}(T) = e^{\mathbf{A}T}\mathbf{X}_0 + \int_0^T e^{\mathbf{A}(T-\sigma)}\mathbf{B}u(\sigma)d\sigma. \tag{3}$$

Hence for $\mathbf{X}(T) = \mathbf{X}_\mathrm{F} = (0, 0)'$, it requires that the control $u(t)$ satisfies

$$\int_0^T u(\sigma)\cos(\omega\sigma)d\sigma = -\frac{\pi}{2}, \tag{4}$$

$$\int_0^T u(\sigma)\sin(\omega\sigma)d\sigma = 0, \tag{5}$$

or equivalently

$$\int_0^T u(t)\, e^{i\omega t}dt = -\frac{\pi}{2}, \tag{6}$$

in order to complete the desired transfer.

We know from linear systems theory [1] that the system (1) is controllable if $\omega \neq 0$, and therefore there exists at least one control $u(t)$ that will accomplish the transfer from $\mathbf{X}_0$ to $\mathbf{X}_\mathrm{F}$. It is also well-known that the minimum-energy control, $u^*(t)$, that achieves the desired transfer while minimizing the total energy, i.e., $\int_0^T u'(t)u(t)dt$, takes the form

$$u^*(t) = \mathbf{B}'e^{-\mathbf{A}t}\mathbf{W}^{-1}\xi, \tag{7}$$

where $\xi = e^{-\mathbf{A}T}\mathbf{X}_{\mathrm{F}} - \mathbf{X}_0$ and $\mathbf{W}$ is the so-called controllability Gramian, given by

$$\mathbf{W} = \int_0^T e^{-\mathbf{A}t}\mathbf{B}\mathbf{B}' e^{-\mathbf{A}'t} dt = \begin{bmatrix} \frac{T}{2} + \frac{\sin(2\omega T)}{4\omega} & -\frac{\sin^2(\omega T)}{2\omega} \\ -\frac{\sin^2(\omega T)}{2\omega} & \frac{T}{2} - \frac{\sin(2\omega T)}{4\omega} \end{bmatrix}. \tag{8}$$

For example, if the frequency of the spring is $\omega = 3$, then the minimum-energy control of duration π is

$$u^*(t) = \frac{3\pi\big[\sin(3t) + \sin(6T - 3t) - 6T\cos(3t)\big]}{\cos(6T) + 18T^2 - 1} = -\cos(3t), \tag{9}$$

for $t \in [0, \pi]$.

Supplementary Note 2: Mapping Spins into Springs

The evolution of the bulk magnetization of a sample of nuclear spins immersed in an external magnetic field follows the Bloch equations [2], given by

$$\frac{d}{dt}\begin{bmatrix} x(t) \\ y(t) \\ z(t) \end{bmatrix} = \begin{bmatrix} 0 & -\omega & u(t) \\ \omega & 0 & -v(t) \\ -u(t) & v(t) & 0 \end{bmatrix}\begin{bmatrix} x(t) \\ y(t) \\ z(t) \end{bmatrix}, \tag{10}$$

where $\mathbf{M}(t) \doteq (x(t), y(t), z(t))'$ is the magnetization vector and $u = -\gamma B_{1y}$ and $v = -\gamma B_{1x}$ are the applied RF fields in the y and x axis, respectively. As before, let $m(t) = x(t) + iy(t)$ be the complex transverse magnetization and $\bar{\alpha}(t) = u(t) - iv(t)$ (the complex conjugate of α) be the irradiating RF field the Bloch equations may then be written in the complex form, that is,

$$\dot{m} = i\omega m + z\bar{\alpha}, \tag{11}$$

$$\dot{z} = -\frac{1}{2}(m\alpha + \bar{m}\bar{\alpha}), \tag{12}$$

where $\bar{\alpha}$ denotes the complex conjugate of α.

Dynamic Mapping Between Spin and Spring

Defining $f : [0, T] \to \mathbb{C}$ by

$$f(t) = \frac{m(t)}{a(t) + z(t)}, \tag{13}$$

where $a(t)$ is a complex-valued function over $[0, T]$, then (11) and (12) can be transformed into the following Riccati equation,

$$\dot{f} = i\omega f + \frac{1}{2}\alpha f^2 + \frac{1}{2}\bar{\alpha} + \frac{\bar{\alpha}(1 - a^2) - 2m\dot{a}}{2(a + z)^2}, \tag{14}$$

in which we used $m\bar{m} + z^2 = 1$ since $\mathbf{M}(t)$ is conventionally a unit vector. Without loss of generality, we consider driving the Bloch equations as in (10) with one control, letting $v = 0$ (in later notes we consider the case with two controls), and consider the canonical state transfer, equivalent to a 90° pulse, from the initial state $\mathbf{M}(0) = (1, 0, 0)'$ in the transverse plane (i.e., $m(0) = 1$ and $z(0) = 0$) to a final state $\mathbf{M}(T) = (0, 0, 1)'$ (i.e., $m(T) = 0$ and $z(T) = 1$). Thus, we have $\alpha = \bar{\alpha} = u$, and (14) becomes

$$\dot{f} = i\omega f + \frac{1}{2}uf^2 + \frac{1}{2}u + \frac{u(1 - a^2) - 2m\dot{a}}{2(a + z)^2}. \tag{15}$$

If we choose the function $a(t)$ such that

$$\frac{u(1-a^2)-2m\dot{a}}{2(a+z)^2}=\frac{e^{2i\omega t}-1}{2}u, \tag{16}$$

with the initial condition $a(0)=1$, namely, $a(t)$ satisfies the Riccati equation

$$\dot{a}=-\frac{u\beta}{2m}a^2-\frac{uz(\beta-1)}{m}a+\frac{u(1+z^2-z^2\beta)}{2m}, \quad a(0)=1, \tag{17}$$

where $\beta=e^{2i\omega t}$, then f follows

$$\dot{f}=i\omega f+\frac{1}{2}uf^2+\frac{1}{2}\beta u, \tag{18}$$

with $f(0)=1$ since $m(0)=1$, $z(0)=0$, and $a(0)=1$. Now, let $g(t)=e^{-i\omega t}f(t)$, that is, f in the rotating frame with respect to ω, then we obtain

$$\dot{g}=\frac{1}{2}(g^2+1)\,ue^{i\omega t}. \tag{19}$$

By the separation of variables, we have

$$\int_{g_0}^{g_1}\frac{2dg}{g^2+1}=\int_0^T u\,e^{i\omega t}dt, \tag{20}$$

where $g_0=g(0)=1$ and $g_1=g(T)$. If the control function satisfies (6), then we have

$$\int_{g_0}^{g_1}\frac{2dg}{g^2+1}=-\frac{\pi}{2}, \tag{21}$$

which, by contour integration, leads to $g_1=g(T)=0$, and hence $f(T)=0$. More specifically, the contour integration and the residue theorem [3] applied to (20) with the condition (6) gives

$$\begin{aligned}\int_1^{c+id}\frac{2dg}{g^2+1}&=2\pi i\,w_1\,\mathrm{Res}(\frac{2}{g^2+1},i)+2\pi i\,w_2\,\mathrm{Res}(\frac{2}{g^2+1},-i)-\int_d^0\frac{2idy}{(c+iy)^2+1}-\int_c^1\frac{2dx}{x^2+1},\\&=2\pi(w_1-w_2)+\int_0^d\frac{2idy}{(c+iy)^2+1}+2\tan^{-1}c-\frac{\pi}{2},\\&=2\pi(w_1-w_2)+2\tan^{-1}(c+id)-\frac{\pi}{2}=-\frac{\pi}{2},\end{aligned} \tag{22}$$

where $g_1=c+id$, $c,d\in\mathbb{R}$, and w_1 and w_2 are the respective winding numbers of the poles i and $-i$. The above equation is reduced, by the logarithmic form of the arctangent function, to

$$\frac{i}{2}\ln\left[\frac{c^2+(1+d)^2}{c^2+(1-d)^2}\right]+\tan^{-1}\left[\frac{2c}{1-c^2-d^2}\right]=0, \tag{23}$$

Therefore, we have

$$\tan^{-1}\left[\frac{2c}{1-c^2-d^2}\right]=0 \quad \text{and} \quad \ln\left[\frac{c^2+(1+d)^2}{c^2+(1-d)^2}\right]=0, \tag{24}$$

which lead to $c=0$ and $d=0$ and give $g_1=0$.

This result shows that a control that steers the spring modeled in (1) from $(\pi/2, 0)'$ to $(0, 0)'$, which satisfies (6), will drive the function f from $f(0) = 1$ to $f(T) = 0$. It follows that this steering control is a candidate $\frac{\pi}{2}$ pulse for the spin system, because, from (13),

$$\begin{cases} (m(0), z(0))' = (1, 0)', & \text{when } f(0) = 1 \text{ with } a(0) = 1, \\ (m(T), z(T))' = (0, 1)', & \text{when } f(T) = 0, \text{ provided } a(T) < \infty, \end{cases} \tag{25}$$

which correspond to $\mathbf{M}(0) = (1, 0, 0)'$ and $\mathbf{M}(T) = (0, 0, 1)'$, respectively. Note that when $m(T) = 0$, $a(t)$ may have singular solutions at $t = T$, which will be analyzed in Supplementary Note 3. An example minimum-energy control $u^*_{\pi/2}(t) = -\cos(3t)$, as expressed in (9) for $\omega = 3$ and $T = \pi$, that steers the spring from $\mathbf{X}_0 = (\pi/2, 0)'$ to $\mathbf{X}_\mathrm{F} = (0, 0)'$, and the evolutions of $a(t)$, $f(t)$, and $g(t)$ as described in (17), (18), and (19), respectively, resulting from this optimal control are illustrated in Supplementary Figure 1.

Similarly, the same concept introduced above can be adopted to design a π pulse, which is realized by constructing a control that steers the spring between $(\pi, 0)'$ and $(0, 0)'$ or simply by concatenating a $\pi/2$ pulse with its time-reversed version. The minimum-energy π pulse, $u^*_\pi = -2\cos(3t)$, $t \in [0, \pi]$, and the resulting trajectories for the spring and the spin of $\omega = 3$ and $T = \pi$ are illustrated in Supplementary Figure 2.

Supplementary Note 3: Regular Singular Solutions of $a(t)$

The Riccati equation for a as in (17) can be reduced to a second order linear ordinary differential equation of the form [4]

$$\ddot{A} - R(t)\dot{A} + S(t)A = 0, \tag{26}$$

where

$$R(t) = -\frac{uz(\beta - 1)}{m} + \frac{\dot{(u\beta)}}{u\beta} - \frac{\dot{m}}{m}, \tag{27}$$

$$S(t) = -\frac{u^2\beta(1 + z^2 - z^2\beta)}{4m^2}, \tag{28}$$

with the relation

$$\frac{\dot{A}}{A} = a\frac{u\beta}{2m}. \tag{29}$$

Note that $\dot{A}(0) = \frac{1}{2}u(0)A(0)$ since $\beta(0) = 1$, $a(0) = 1$, and $m(0) = 1$. In addition to the case $m(T) = 0$ as in (25), the other possibility for $f(T) = \frac{m(T)}{a(T)+z(T)} = 0$ to hold is when $a(T) = \infty$. This occurs when the coefficients of (26), i.e., $R(t)$ and $S(t)$ in (27) and (28), respectively, develop singularities at $t = T$, which in turn occurs when $m(T) = 0$. Let

$$L_1 = \lim_{t \to T} \big\{(T - t)R(t)\big\}, \tag{30}$$

$$L_2 = \lim_{t \to T} \big\{(T - t)^2 S(t)\big\}. \tag{31}$$

If both the limits L_1 and L_2 are finite, then $t = T$ is a regular singular point. Note that since $R(t)$ and $S(t)$ are determined by the control variable u, one may expect to design a feasible control such that $t = T$ is regular singular when $m(T) = 0$. In this case, we can write (26) as

$$\ddot{A} - \frac{P(t)}{t}\dot{A} + \frac{Q(t)}{t^2}A = 0, \tag{32}$$

where $R(t) = P(t)/t$ and $S(t) = Q(t)/t^2$. Without loss of generality, we may assume that this regular singular point is transformed to $t = 0$; or, equivalently, we redefine the time axis by letting $t = t - T$, so now $t = 0$ represents $t = T$. Then, there exists a solution to (32) around the regular singular point, $t = 0$, of the form (a Frobenius series) [4],

$$A(t) = \sum_{k=0}^{\infty} \alpha_k t^{k+r}, \quad \alpha_0 \neq 0, \tag{33}$$

where r is a definite (real or complex) constant, and this solution is valid in some interval $0 < |t| < \rho$, $\rho > 0$. Plugging (33) into (32) yields

$$\sum_{k=0}^{\infty}(k+r)(k+r-1)\alpha_k t^{k+r-2} - \sum_{k=0}^{\infty} P(t)(k+r)\alpha_k t^{k+r-2} + \sum_{k=0}^{\infty} Q(t)\alpha_k t^{k+r-2} = 0. \tag{34}$$

Taking the series expansions for $P(t) = \sum_{i=0}^{\infty} p_i t^i$ and $Q(t) = \sum_{i=0}^{\infty} q_i t^i$ in (34) and collecting the coefficient of t^{k+r-2} gives

$$\sum_{k=0}^{\infty} \Big[(k+r)(k+r-1) - P(t)(k+r) + Q(t)\Big]\alpha_k t^{k+r-2} = 0, \tag{35}$$

which, for $k = 0$, leads to the indicial equation,

$$F(r) = r(r-1) - p_0 r + q_0 = 0, \tag{36}$$

since (35) holds for all $k = 0, 1, 2, \ldots$, and $\alpha_0 \neq 0$.

Furthermore, p_0 and q_0 in (36) can be represented in terms of the control variable and the state of the spin system. We first recall that $m(T) = 0$, so we have $x(T) = 0$, $y(T) = 0$, and $z(T) = 1$. Now, let's take the Taylor expansions for the state and control functions around the regular singular point, which is now $t = 0$, to get

$$u = u_0 + u_1 t + u_2 t^2 + \cdots, \tag{37}$$

$$x = x_1 t + x_2 t^2 + x_3 t^3 + \cdots, \tag{38}$$

$$z = 1 + z_1 t + z_2 t^2 + \cdots, \tag{39}$$

where $x_0 = x(T) = 0$ and $z_0 = z(T) = 1$. Also, because, from the Bloch equations in (10) with $v = 0$, $\dot{y} = \omega x$, we obtain

$$y(t) = y_0 + \int_0^t \omega x(\sigma) d\sigma = \int_0^t \omega(x_1\sigma + x_2\sigma^2 + \cdots) d\sigma = \frac{\omega x_1}{2} t^2 + \frac{\omega x_2}{3} t^3 + \cdots, \tag{40}$$

in which $y_0 = y(T) = 0$, and hence

$$m = x + iy = x_1 t + (x_2 + i\frac{\omega x_1}{2})t^2 + (x_3 + i\frac{\omega x_2}{3})t^3 + \cdots. \tag{41}$$

In addition, integrating the z-component in the Bloch equations, which obeys $\dot{z} = -ux$, and employing (37) and (38) yields

$$z = 1 - \frac{u_0 x_1}{2} t^2 - \frac{u_0 x_2 + u_1 x_1}{3} t^3 - \frac{u_0 x_3 + u_1 x_2 + u_2 x_1}{4} t^4 - \cdots. \tag{42}$$

Using the definitions in (27) and (28) and the relations $R(t) = P(t)/t = \sum_{i=0}^{\infty} p_i t^i/t$ and $S(t) = Q(t)/t^2 = \sum_{i=0}^{\infty} q_i t^i/t^2$ together with the expressions derived in (37), (39), (41), we obtain

$$p_0 = \frac{u_0(1-\beta_0)}{x_1} - 1, \quad q_0 = \frac{u_0^2\beta_0(\beta_0 - 2)}{4x_1^2}, \tag{43}$$

where $\beta = e^{2i\omega t} = \beta_0 + \beta_1 t + \beta_2 t^2 + \cdots$ with $\beta_0 = \beta(T) = e^{2i\omega T}$. Therefore, the indicial equation associated with the second-order ordinary differential equation (26) is given by

$$F(r) = r^2 - \frac{u_0(1-\beta_0)}{x_1} r + \frac{u_0^2\beta_0(\beta_0-2)}{4x_1^2} = 0. \tag{44}$$

Control Synthesis for Regular Singular Solutions

Given the expression in (44), we can design controls (pulses) and choose their durations to manipulate the indicial equation and thus the regular singular solution of (26) around the regular singular point, that is, $t = 0$. The indicial equation in (44) has two roots, given by

$$r_1 = \frac{u_0(2-\beta_0)}{2x_1}, \qquad r_2 = \frac{-u_0\beta_0}{2x_1}. \tag{45}$$

Recall that $x_1 = \dot{x}(T)$ and $u_0 = u(T)$. We may now show that $a(T)$ is finite, provided $x_1 \neq 0$ and $u_0 \neq 0$.

Lemma 1 *If $u_0 = u(T) \neq 0$ and the resulting x-trajectory has a non-vanished derivative at $t = T$, i.e., $x_1 = \dot{x}(T) \neq 0$, then $a(T) < \infty$.*

Proof. Because $u_0 \neq 0$ and $x_1 \neq 0$, we have $r_1 \neq 0$, $r_2 \neq 0$, and $r_1 - r_2 = \frac{u_0}{x_1}$ from (45). The regular singular solution to the differential equation (26) depends on the relation of $r_1 - r_2$, which leads to two cases.

Case I: $|\mathbf{r_1} - \mathbf{r_2}| = \mathbb{Z}^+$. We first examine the situation when $r_1 > r_2$, i.e., $u_0 = \zeta x_1$, where ζ is a positive integer. Then, the equation (26) has two nontrivial linearly independent solutions of the form

$$A_1(t) = t^{r_1} h_1(t) \tag{46}$$

where $h_1(t) = \sum_{i=0}^{\infty} c_i t^i$ with $c_0 \neq 0$, and

$$A_2(t) = t^{r_2} h_2(t) + cA_1(t)\ln t, \tag{47}$$

where $h_2(t) = \sum_{i=0}^{\infty} d_i t^i$ with $d_0 \neq 0$, and c is a constant that may or may not be 0. The general solution of (26) is $A(t) = k_1 A_1(t) + k_2 A_2(t)$, where $k_1, k_2 \in \mathbb{R}$. Then, we can compute the limit

$$\begin{aligned}
\lim_{t\to 0} \frac{t\dot{A}(t)}{A(t)} &= \lim_{t\to 0} \frac{t\big[k_1\dot{A}_1 + k_2\dot{A}_2\big]}{k_1A_1 + k_2A_2} \\
&= \lim_{t\to 0} \frac{tk_1(r_1h_1t^{r_1-1} + \dot{h}_1t^{r_1}) + tk_2c(r_1h_1t^{r_1-1} + \dot{h}_1t^{r_1})\ln t + k_2ch_1t^{r_1} + k_2r_2h_2t^{r_2} + k_2\dot{h}_2t^{r_2+1}}{k_1h_1t^{r_1} + k_2ch_1t^{r_1}\ln t + k_2h_2t^{r_2}} \\
&= \lim_{t\to 0} \frac{k_1(r_1h_1t^{r_1-r_2} + \dot{h}_1t^{r_1+1-r_2}) + k_2c(r_1h_1t^{r_1-r_2} + \dot{h}_1t^{r_1+1-r_2})\ln t + k_2ch_1t^{r_1-r_2} + k_2r_2h_2 + k_2\dot{h}_2t}{k_1h_1t^{r_1-r_2} + k_2ch_1t^{r_1-r_2}\ln t + k_2h_2} \\
&= r_2,
\end{aligned} \tag{48}$$

provided $k_2 \neq 0$. By the transformation $a(t) = \frac{2m\dot{A}}{u\beta A}$ from (29) with (41) and (48), we obtain the limit of $a(t)$,

$$\lim_{t\to 0} a(t) = \lim_{t\to 0} \frac{2(x_1 t + \cdots)\dot{A}}{(u_0 + u_1 t + \cdots)(\beta_0 + \beta_1 t + \cdots)A} = \frac{2x_1 r_2}{u_0\beta_0} = \frac{2x_1(\frac{-u_0\beta_0}{2x_1})}{u_0\beta_0} = -1, \tag{49}$$

where we employed (37) and (38).

If $r_2 > r_1$, i.e., $u_0 = -\zeta x_1$ with $\zeta \in \mathbb{Z}^+$, then the two linearly independent solutions of (26) are of the form $A_1(t) = t^{r_2} h_1(t)$ and $A_2(t) = t^{r_1} h_2(t) + cA_1(t)\ln t$. Similar calculations as in (48) give $\lim_{t\to 0} \frac{t\dot{A}(t)}{A(t)} = r_1$ and thus

$$\lim_{t\to 0} a(t) = \frac{2x_1 r_1}{u_0\beta_0} = \frac{2x_1(\frac{u_0(2-\beta_0)}{2x_1})}{u_0\beta_0} = \frac{2-\beta_0}{\beta_0}. \tag{50}$$

Combining the above two cases, we conclude that

$$\lim_{t\to 0} a(t) = \begin{cases} \frac{2-\beta_0}{\beta_0}, & \text{if } \frac{u_0}{x_1} < 0, \\ -1, & \text{if } \frac{u_0}{x_1} > 0, \end{cases} \tag{51}$$

where $\beta_0 = \beta(T) = e^{2i\omega T}$. Furthermore, let $\eta = \frac{2-\beta_0}{\beta_0}$, then we have $|\eta| = |2-\beta_0| = \sqrt{5 - 4\cos(2\omega T)}$. Therefore, η is bounded by $1 \leq |\eta| \leq 3$, and $|\eta| = 1$ when $T = n\pi/\omega$, and $|\eta| = 3$ when $T = (2n+1)\pi/(2\omega)$, where $n = 0, 1, 2, \ldots$.

Case II: $\mathbf{r_1 - r_2 \neq 0}$ or $|\mathbf{r_1 - r_2}| \neq \mathbb{Z}^+$. In this case, the equation (26) has a general solution of the form $A(t) = k_1 A_1(t) + k_2 A_2(t)$, where

$$A_1(t) = t^{r_1} h_1(t), \tag{52}$$

in which $h_1(t) = \sum_{i=0}^{\infty} c_i t^i$ with $c_0 \neq 0$, and

$$A_2(t) = t^{r_2} h_2(t), \tag{53}$$

in which $h_2(t) = \sum_{i=0}^{\infty} d_i t^i$ with $d_0 \neq 0$. Similar calculations as described in Case I lead to the same result, that is,

$$\lim_{t\to 0} a(t) = \begin{cases} \frac{2-\beta_0}{\beta_0}, & \text{if } \frac{u_0}{x_1} < 0, \\ -1, & \text{if } \frac{u_0}{x_1} > 0, \end{cases} \tag{54}$$

so that $1 \leq |\lim_{t\to 0} a(t)| \leq 3$. □

To conclude, in this note, we illustrate that if the limits L_1 and L_2 defined in (30) and (31), respectively, exist, then the second-order differential equation A in (26) has a regular singular solution at $t = T$ and $a(T)$ is finite. This implies that when $f(T) = 0$, it must be $m(T) = 0$ as desired. Moreover, the regular singular solution depends on the control design, because u_0 and x_1, which results from u_0, determine the indicial equation. Ideally, one can design an admissible control input, satisfying the integral condition in (6), such that $u_0 = u(T) \neq 0$, $x_1 = \dot{x}(T) \neq 0$, and $t = T$ is a regular singular point. With these conditions fulfilled, the control function $u(t)$ for $t \in [0, T]$ drives the spin from $e_1 = (1, 0, 0)'$ to $e_3 = (0, 0, 1)'$, and hence the time-reversed and sign-inverted control function $\tilde{u}(t) = -u(T - t)$ for $t \in [0, T]$ is a $\pi/2$ pulse that excites the spin from the equilibrium state e_3 to the excited state e_1.

Asymptotic Exactness

A curious, but important, characteristic of the spin trajectory corresponding to the minimum energy control pulse - and, therefore, a characteristic of the mapping between spin and spring - is that the performance (the closeness of the final z magnetization to 1) approaches 1 as the product ωT increases, i.e., as either or both the frequency of the Bloch system or the duration of the minimum energy pulse increases (see Supplementary Figure 3). The numerical experiments shown in Supplementary Figure 3 indicate that as the value of ωT increases, $a(T)$ asymptotically approaches being a regular singular point, which yields finite values of $a(T)$ and forces the asymptotic behavior $z(T) \to 1$. Therefore, the framework we establish in this paper quantifies sufficient conditions for asymptotic exactness. In some contexts our approach may yield arbitrarily exact excitation pulses; in other applications which require restricted durations, our findings provide almost exact excitation pulses with a guide for the relationship between performance, duration, and frequency.

Supplementary Note 4: An Alternative Dynamic Mapping

We now discuss an alternative dynamic mapping scenario that permits the use of two controls in the analysis and provides a different perspective on the dynamic connection between the spring and the spin, in particular, delivering an explanation for the asymptotic behavior of the performance $z(T)$, through the asymptotic behavior of $a(T)$. If, instead of using (17), $a(t)$ is chosen such that

$$\frac{1}{2}\bar{\alpha} + \frac{\bar{\alpha}(1-a^2) - 2m\dot{a}}{2(a+z)^2} = 0, \tag{55}$$

with the initial condition $a(0) = 1$, namely, $a(t)$ satisfies the differential equation

$$\dot{a} = \frac{\bar{\alpha}}{2m}\left(2za + z^2 + 1\right), \quad a(0) = 1, \tag{56}$$

then f follows

$$\dot{f} = i\omega f + \frac{1}{2}\alpha f^2, \tag{57}$$

with $f(0) = 1$ since $m(0) = 1$, $z(0) = 0$, and $a(0) = 1$. Similar to the derivation in Supplementary Note 2, let $g(t) = e^{-i\omega t} f(t)$, then it follows that

$$\dot{g} = \frac{1}{2}\, g^2\, \alpha\, e^{i\omega t}, \tag{58}$$

and, furthermore,

$$\int_{g_0}^{g_1} \frac{2}{g^2}\, dg = \int_0^T \alpha\, e^{i\omega t} dt, \tag{59}$$

where $g_0 = g(0) = 1$ and $g_1 = g(T)$. One can show that the line integral $\int_\gamma \frac{2}{g^2} dg$ is independent of the path γ that starts with $g_0 = 1$ and ends at $g_1 = c + id$ with $c, d \in \mathbb{R}$. Let c_1 and c_2 be two non-homotopic loops in $\mathbb{C}\backslash\{0\}$, and let D_1 and D_2 denote the regions inside C_1 and C_2, respectively. Without loss of generality, we assume that $0 \notin D_1$ but $0 \in D_2$. Therefore, C_1 is homologous to 0, and the integrand $2/g^2$ is analytic on D_1. Hence, we have

$$\int_{C_1} \frac{2}{g^2} dg = 0, \tag{60}$$

and, in addition, the Cauchy's integral formula gives

$$\int_{C_2} \frac{2}{g^2} dg = \frac{n(C_2,0)}{2\pi i} \frac{d}{dg}\Big|_{g=0} 2 = 0, \tag{61}$$

where $n(C_2, 0)$ denotes the winding number of C_2 with respect to 0. As a result, the integral $\int_C \frac{2}{g^2} dg = 0$ over any loop C, and thus it is path-independent.

Now, consider a path γ along the real axis from $g_0 = 1$ to c and then align the imaginary axis from c to $g_1 = c + id$, then the line integral

$$\int_\gamma \frac{2}{g^2} dg = \int_1^c \frac{2}{x^2} dx + \int_0^d \frac{2}{(c+iy)^2} d(iy) = 2\left(1 - \frac{c}{c^2+d^2} + i\frac{d}{c^2+d^2}\right). \tag{62}$$

If the control function satisfies (6), then we obtain, using (59) and (62),

$$d = 0, \quad 2(c-1)/c = -\pi/2. \tag{63}$$

This gives

$$g_1 = \frac{4}{4+\pi}, \tag{64}$$

and

$$f(T) = \frac{4}{4+\pi} e^{i\omega t}. \tag{65}$$

If, in addition, the control drives $a(T) \to -1$, then

$$\frac{4}{4+\pi} e^{i\omega T} = f(T) = \frac{m(T)}{a(T) + z(T)} = \frac{x_1 + iy_1}{z_1 - 1}, \tag{66}$$

where $x_1 = x(T) \in \mathbb{R}$, $y_1 = y(T) \in \mathbb{R}$, and $z_1 = z(T) \in \mathbb{R}$, with $z_1^2 = 1 - x_1^2 - y_1^2$. Solving this equation yields two real solutions

$$\begin{aligned} &(i) \quad x_1 = 0, \quad y_1 = 0, \quad z_1 = 1 \\ &(ii) \quad x_1 = -\frac{8(4+\pi)\cos(\omega T)}{\pi(8+\pi)+32}, \quad y_1 = -\frac{8(4+\pi)\sin(\omega T)}{\pi(8+\pi)+32}, \quad z_1 = -\frac{\pi(8+\pi)}{\pi(8+\pi)+32}, \end{aligned} \tag{67}$$

and solution (ii) can be omitted since $z(T) > 0$ by the application of the control u that satisfies (6). This can be seen by considering the case of $\omega = 0$, where following the control satisfying (6), the spin is steered from $(1, 0, 0)'$ to $(0, 0, 1)'$ at time T. A continuity argument in ω may lead to the required conclusion.

It follows that this steering control is a candidate $\frac{\pi}{2}$ pulse for the spin system, because, from (13),

$$\begin{cases} (m(0), z(0))' = (1, 0)', & \text{when } f(0) = 1 \text{ with } a(0) = 1, \\ (m(T), z(T))' = (0, 1)', & \text{when } f(T) = \frac{4}{4+\pi} e^{i\omega T}, \text{ with } a(T) = -1, \end{cases} \tag{68}$$

which correspond to $\mathbf{M}(0) = (1, 0, 0)'$ and $\mathbf{M}(T) = (0, 0, 1)'$, respectively.

Numerical calculations show that following the example minimum-energy control $u^*_{\pi/2}(t) = -\cos(3t)$ expressed in (9) for $\omega = 3$ and $T = \pi$, the final state $a(T) \to -1$ asymptotically with respect to the final time T - the length of the pulse - (see Supplementary Figure 3), and the spin is steered from $(1, 0, 0)'$ asymptotically to $(0, 0, 1)'$. The evolutions of $a(t)$, $f(t)$, and $g(t)$ as

described in (56), (57), and (58), respectively, resulting from this optimal control are illustrated in Supplementary Figure 4.

Moreover, we observe empirical evidence (see Supplementary Figure 5) that the correlation between the spin rotation angle and the initial state of the harmonic oscillator seem to be an exact correspondence. We have proven that driving the harmonic oscillator from $(\frac{\pi}{2}, 0)'$ to $(0, 0)'$ achieves a $\frac{\pi}{2} = 90°$ rotation of the spin vector. By simulation we see that driving the spring from $(\frac{\pi}{4}, 0)'$ instead corresponds to a rotation of the spin vector by $\frac{\pi}{4} = 45°$. Thus, we conjecture that any total rotation γ (in the x-z plane) can be reached by satisfying,

$$2\left(\frac{c-1}{c}\right) = -\gamma \qquad \Rightarrow \qquad g_1 = \frac{2}{2+\gamma}, \tag{69}$$

according to (63), where $(\gamma, 0)'$ is the initial state of the spring and $x_0 = \sin(\gamma)$ is the initial x state of the spin.

Supplementary Note 5: Complex Representation of the Spring Driven by Two Controls

In the preceding note, the alternative derivation provided a proof that allowed both controls of the spin (u and v) to be nonzero, i.e., $\alpha = u + iv$ was left general. This provides an opportunity to connect the full Bloch equations of the spin to the case where the spring is driven by two controls as well. This provides a clearer explanation of the correspondence between spin and spring. We consider the system (1) with a second control that directly forces the position term of the harmonic oscillator,

$$\frac{d}{dt}\begin{bmatrix}\tilde{x}(t)\\ \tilde{y}(t)\end{bmatrix} = \begin{bmatrix}0 & -\omega\\ \omega & 0\end{bmatrix}\begin{bmatrix}\tilde{x}(t)\\ \tilde{y}(t)\end{bmatrix} + \begin{bmatrix}1 & 0\\ 0 & 1\end{bmatrix}\begin{bmatrix}u(t)\\ v(t)\end{bmatrix}. \tag{70}$$

A compact expression for (70) is provided by complex notation with $\tilde{m}(t) = \tilde{x}(t) + i\tilde{y}(t)$, $\alpha(t) = u(t) + iv(t)$,

$$\dot{\tilde{m}} = i\omega\tilde{m} + \alpha. \tag{71}$$

We can develop analogous conditions for the transfer from $\tilde{m}(0) = \frac{\pi}{2}$ to $\tilde{m}(T) = 0$ since,

$$\tilde{m}(T) = 0 = e^{i\omega T}\frac{\pi}{2} + \int_0^T e^{i\omega(T-t)}\alpha(t)\; dt$$

$$-\frac{\pi}{2} = \int_0^T \alpha(t)e^{-i\omega t}\; dt \tag{72}$$

$$= \underbrace{\int_0^T [u(t)\cos(\omega t) + v(t)\sin(\omega t)]\; dt}_{=-\frac{\pi}{2}} + i\underbrace{\int_0^T [v(t)\cos(\omega t) - u(t)\sin(\omega t)]\; dt}_{=0}. \tag{73}$$

We observe that (72) (spring, two controls) differs from (6) (spring, one control) by the sign of the exponential term. More importantly, we note that (72) differs from (59) (spin, two controls). This means that in the general two control case, the conditions for spin and spring no longer coincide without additional criteria. We observe that the single control case, in which $v(t) = 0$, is a simple way (sufficient condition) to make these two expressions equivalent. However, it is instructive to

identify the necessary and sufficient conditions for (72) and (59) to be equivalent in the general case when two controls are allowed, i.e., establish when

$$\underbrace{\int_0^T \alpha(t)e^{-i\omega t}\, dt}_{\text{spring (72)}} = \underbrace{\int_0^T \alpha(t)e^{i\omega t}\, dt}_{\text{spin (59)}}\,. \tag{74}$$

These integral conditions coincide if and only if

$$\int_0^T u(t)\sin(\omega t)\, dt = 0, \tag{75}$$

$$\int_0^T v(t)\sin(\omega t)\, dt = 0. \tag{76}$$

Again, in the case of a single control, $v(t) \equiv 0$ guarantees (76) and the design of $u(t)$ to satisfy (5) guarantees (75). In the general case, (75) and (76) identify the additional criteria required to design controls that simultaneously drive the spin and spring.

The general minimum energy control that achieves the desired transformation using two controls can be computed as

$$\alpha(t) = e^{-i\omega t}W^{-1}\left(-\frac{\pi}{2}\right) = -\frac{\pi}{2T}e^{-i\omega t} = \underbrace{-\frac{\pi}{2T}\cos(\omega t)}_{u(t)} + i\Big(\underbrace{-\frac{\pi}{2T}\sin(\omega t)}_{v(t)}\Big), \tag{77}$$

where the controllability Gramian is given by

$$W = \int_0^T e^{-i\omega t}e^{i\omega t}\, dt = T, \tag{78}$$

since in the context of complex numbers A' in the previous definition becomes $A^\dagger$, i.e., the conjugate transpose of A. However, this minimum energy control differs from the expression in (7) because it assumes two controls, whereas (7) assumes only one control.

We further observe, without a formal proof, that due to the linearity of the spring, we can easily design controls to start from initial states that have nonzero imaginary part (e.g., $\tilde{m}(0) = \frac{\pi}{2\sqrt{2}} + i\frac{\pi}{2\sqrt{2}}$) by independently designing $u(t)$ to accomplish the transfer for the real part, i.e., $\tilde{m}(0) = \frac{\pi}{2\sqrt{2}}$, and $v(t)$ to accomplish the transfer for the imaginary part, i.e., $\tilde{m}(0) = i\frac{\pi}{2\sqrt{2}}$.

For a spin magnetization described in spherical coordinates with radial distance $\rho = 1$, azimuthal angle θ, and polar angle ϕ, the spin cartesian coordinates are $\mathbf{M} = (\cos\theta\sin\phi, \sin\theta\sin\phi, \cos\phi)'$ (simply transforming from spherical to cartesian coordinates). Here ϕ represents the angle that the spin vector must be rotated to bring it to the final state $(0, 0, 1)'$. The case in the manuscript considers a rotation of $\pi/2$. At the end of Supplementary Note 4 we discussed the extension to design a single control that generates an arbitrary flip angle γ along either the x or y axes. Now with two controls we can accommodate any flip angle along any arbitrary direction. The corresponding spring state captures the fact that the overall spin rotation is ϕ, but is shared between the two controls (which is specified by the angle θ): $\tilde{m} = \phi\cos\theta + i\ \phi\sin\theta$. Therefore, while ϕ is the total rotation angle of the spin achieved by both controls applied together, the effective rotation that each control must generate is $\phi\cos\theta$ for the u control and $\phi\sin\theta$ for the v control.

Importantly - and different from the two-control minimum energy control (77) - this independent design of $u(t)$ and $v(t)$ automatically satisfies both (75) and (76). Therefore, when designed

independently in this manner, the combined controls also become controls for the spin (e.g., driving the spin from $\mathbf{M}(0) = (\frac{1}{\sqrt{2}}, \frac{1}{\sqrt{2}}, 0)'$ to $\mathbf{M}(T) = (0, 0, 1)'$). Specifically, a general transfer, i.e., from $\tilde{m}(0) \to \tilde{m}(T)$, can be achieved by designing $u(t)$ and $v(t)$ to satisfy

$$\int_0^T u(t)\cos(\omega t)\ dt = \tilde{m}(T)\cos(\omega T) - \tilde{m}(0), \quad \int_0^T u(t)\sin(\omega t)\ dt = 0, \tag{79}$$

$$\int_0^T v(t)\sin(\omega t)\ dt = 0, \quad \int_0^T v(t)\cos(\omega t)\ dt = -\tilde{m}(T)\sin(\omega T). \tag{80}$$

This approach aligns with the fact that $v(t) = 0$ when the imaginary part of $\tilde{m}(0)$ is zero (the case considered in the main manuscript and first proof). Supplementary Figure 5 depicts an example of such a control and the corresponding state trajectories.

Supplementary Note 6: Validation of the Dynamic Transformation

Because the dynamic transformation $f : [0, T] \to \mathbb{C}$ as in (13) may correspond to an invalid spin trajectory, i.e., a complex-valued trajectory, depending on $a(t)$, which in turn depends on the applied control $u(t)$, it is then essential to examine conditions under which $f(t)$ with $f(0) = 1$ uniquely determines a Bloch trajectory $(x(t), y(t), z(t))'$ on the unit sphere for $t \in [0, T]$. Let $f(t) = f_1(t) + if_2(t)$ and $a(t) = a_1(t) + ia_2(t)$, and then from (13) we have

$$f_1(t) + if_2(t) = \frac{x(t) + iy(t)}{a_1(t) + ia_2(t) + z(t)}, \tag{81}$$

for all $t \in [0, T]$. Since $(x, y, z)'$ is a Bloch vector, it satisfies, for all $t \in [0, T]$,

$$x^2(t) + y^2(t) + z^2(t) = 1. \tag{82}$$

Solving (81) and (82) for $(x, y, z)'$ yields,

$$x_1 = f_1(a_1 + z_1) - a_2 f_2, \quad y_1 = a_2 f_1 + f_2(a_1 + z_1), \quad z_1 = \frac{a_1 - D}{1 + |f|^2} - a_1, \tag{83}$$

or

$$x_2 = f_1(a_1 + z_2) - a_2 f_2, \quad y_2 = a_2 f_1 + f_2(a_1 + z_2), \quad z_2 = \frac{a_1 + D}{1 + |f|^2} - a_1, \tag{84}$$

where $D = \sqrt{-a_2^2\,|f|^4 + (1 - |a|^2)|f|^2 + 1}$ and where $|f|^2 = f_1^2 + f_2^2$ and $|a|^2 = a_1^2 + a_2^2$. Therefore, it requires that the discriminant is positive i.e.,

$$-a_2^2\,|f|^4 + (1 - |a|^2)|f|^2 + 1 > 0, \tag{85}$$

in order for $(x_i(t), y_i(t), z_i(t))'$ to be a Bloch trajectory. Employing the initial conditions $f(0) = 1$ and $a(0) = 1$, i.e., $f_1(0) = 1$, $f_2(0) = 0$, $a_1(0) = 1$, and $a_2(0) = 0$, the corresponding Bloch trajectory can be uniquely determined in terms of $f(t)$ and $a(t)$, which is $(x_2(t), y_2(t), z_2(t))'$ for $t \in [0, T]$ as in (84), because $(x_2(0), y_2(0), z_2(0))' = (1, 0, 0)'$. Note that the discriminant in (85) must be strictly positive, because if it is equal to zero, then $D = 0$ implies $(x_1, y_1, z_1)' = (x_2, y_2, z_2)'$ for all $t \in [0, T]$, which gives an invalid initial condition $(\frac{1}{2}, 0, -\frac{1}{2})'$. Furthermore, it follows from

(85) that the necessary and sufficient condition for z_2 to be real, and hence $(x_2(t), y_2(t), z_2(t))'$ a valid Bloch trajectory, is

$$0 \leq |f|^2 < \frac{1 - |a|^2 + \sqrt{(1 - |a|^2)^2 + 4a_2^2}}{2a_2^2}, \tag{86}$$

which determines the regime where the dynamic transformation f determines a valid spin trajectory with respect to an applied control field. Finally, we note that if $a(t) \equiv 1$ for all $t \in [0, T]$, then $D = 1$, $z_1 = -1$ (the south pole), and $z_2 = \frac{1-f\bar{f}}{1+f\bar{f}} = \frac{1-|f|^2}{1+|f|^2}$, which is the Stereographic projection.

To illustrate the importance of the bound on the dynamic projection $f(t)$, in Supplementary Figure 6 we plot the time evolution of D for the minimum-energy control and several counterexample controls, which satisfy the integral condition in (6), but not the bound in (86). Note that all controls steer the spring from $(\pi/2, 0)'$ to $(0, 0)'$ but only when (86) is satisfied, as in the case of the minimum energy control, does the control also steer the spin from $(1, 0, 0)'$ to $(0, 0, 1)'$.

Supplementary Note 7: Broadband Pulse Design via Steering Springs

We have shown that the design of excitation and inversion pulses that manipulate the spin magnetization at a single frequency ω can be mapped into a problem of steering springs, for which finding analytical expressions of optimal steering controls is a straightforward manner. It follows from this new finding that a control simultaneously steering a family of springs over a defined bandwidth between $(0, 0)'$ and $(\pi/2, 0)'$ (or $(\pi, 0)'$) is a broadband $\pi/2$ (or π) pulse, respectively.

Analytic Broadband Pulse Design

Analytic broadband pulses can be constructed through studying the steering problem of an ensemble of harmonic oscillators, given by

$$\frac{d}{dt}\begin{bmatrix} x(t,\omega) \\ y(t,\omega) \end{bmatrix} = \begin{bmatrix} 0 & -\omega \\ \omega & 0 \end{bmatrix}\begin{bmatrix} x(t,\omega) \\ y(t,\omega) \end{bmatrix} + \begin{bmatrix} u(t) \\ v(t) \end{bmatrix}, \tag{87}$$

where $\omega \in [\omega_1, \omega_2] \subset \mathbb{R}$. The minimum-energy ensemble control that drives this spring ensemble from an initial state $\mathbf{X}_0(\omega) = (x(0, \omega), y(0, \omega))'$ to a desired final state $\mathbf{X}_{\mathrm{F}}(\omega) = (x(T, \omega), y(T, \omega))'$ at a prescribed time $T < \infty$ and minimizes the total energy of the control fields, i.e., $J = \int_0^T \left[u(t)^2 + v(t)^2\right] dt$, was derived in our previous work, and the mathematical details can be found in [5]. A brief description is provided below in this note.

Let

$$p(t, \omega) = x(t, \omega) + iy(t, \omega),$$
$$\alpha(t) = u(t) + iv(t),$$

the system (87) is then transformed to a complex system, $\dot{p}(t, \omega) = i\omega p(t, \omega) + \alpha(t)$, with the initial state $p(0, \omega) = x(0, \omega) + iy(0, \omega)$. Without loss of generality, we consider the case in which the frequencies are symmetric to the origin, i.e., $\omega \in [-\beta, \beta]$. By the variation of constants formula, we have at time T

$$p(T, \omega) = e^{i\omega T} p(0, \omega) + \int_0^T e^{i\omega(T-\tau)} \alpha(\tau) d\tau, \tag{88}$$

for all $\omega \in [-\beta, \beta]$, and this gives

$$\int_0^T e^{-i\omega\tau}\alpha(\tau)d\tau = e^{-i\omega T}p(T,\omega) - p(0,\omega) \doteq \xi(\omega). \tag{89}$$

Let $H_1 = L_2[0,T]$ and $H_2 = L_2[-B,B]$ be Hilbert spaces over $\mathbb{C}$. Defining the linear operator $L : H_1 \to H_2$ by

$$(L\alpha)(\omega) = \int_0^T e^{-i\omega\tau}\alpha(\tau)d\tau, \tag{90}$$

it follows from (89) and (90) that

$$(L\alpha)(\omega) = \xi(\omega). \tag{91}$$

It can be shown that the operator L is compact and that the minimum norm solution of (91), which corresponds to the minimum-energy control α^*, is given by

$$\alpha^*(t) = \int_{-\beta}^{\beta} e^{i\omega t}\sum_{n=1}^{\infty}\frac{1}{\lambda_n}\langle\xi,\tilde{\phi}_n\rangle\tilde{\phi}_n\, d\omega, \tag{92}$$

where $\tilde{\phi}_n = e^{-i\omega\frac{T}{2}}\frac{\psi_n}{\|\psi_n\|}$ and $\lambda_n = 2\pi\kappa_n$, in which ψ_n are the *prolate spheroidal wave functions (pswf's)* and $\kappa_n > 0$ are the associated eigenvalues [6, 7, 8]. The control α^* that steers the spring ensemble from $p(0,\omega) = \pi/2$ to $p(T,\omega) = 0$ is a broadband $\pi/2$ pulse and that drives the ensemble from $p(0,\omega) = \pi$ to $p(T,\omega) = 0$ is a broadband π pulse. A truncated optimal control $\alpha_N(t) = \int_{-\beta}^{\beta} e^{i\omega t}\sum_{n=1}^{N(\varepsilon)}\frac{1}{\lambda_n}\langle\xi,\tilde{\phi}_n\rangle\tilde{\phi}_n\, d\omega$, will steer the ensemble (87) from $p(0,\omega)$ to be within an ε-neighborhood of the desired final state, namely, $\mathcal{B}_\varepsilon\big(p_{\rm F}(\omega)\big) = \{h \in \mathbf{H}_2 : \|p_{\rm F}(\omega) - h(\omega)\|_2 < \varepsilon\}$ at time T, where the size of ε defines the tolerance of the pulse performance.

Numerical Synthesis of Broadband Pulses

Note that the pswf's can be approximated by the *discrete prolate spheroidal sequences (dpss's)*, denoted $\{v_{t,k}(N,W)\}$, which are defined via the solution to the following algebraic equation [7, 9]

$$\sum_{t'=0}^{N-1}\frac{\sin\left[2\pi W(t-t')\right]}{\pi(t-t')}v_{t',k}(N,W) = \lambda_k(N,W)v_{t,k}(N,W),$$

where $0 < W < \frac{1}{2}$ and $t = 0, 1, \ldots, N-1$. The minimum-energy ensemble control α^* as in (92) and its truncation $\alpha_N(t)$ can be easily calculated numerically using the discrete prolate spheroidal sequences in most scientific programming tools, e.g., the MATLAB command "dpss". A broadband π pulse that produces uniform excitation over the designed bandwidth is shown in Supplementary Figure 7. In addition, this optimal control can also be effectively obtained via finding the minimum-norm solution to the integral equation (90) using the recently developed SVD-based algorithm [10].

Design of Constrained Broadband Pulses

In practice, the amplitude or power of RF pulses may be limited. Such constrained broadband pulse design can be formulated as a minimization problem, where we wish to steer the spring ensemble from $p(0,\omega) = \pi/2$ or $p(0,\omega) = \pi$ to $p_{\rm F}(\omega) = 0$, given by

$$\min_{u,v} \quad \int_{-B}^{B} \| \, p(T,\omega) - p_{\rm F}(\omega) \, \|^2 \, d\omega, \tag{93}$$

$$\text{s.t.} \quad u^2(t) + v^2(t) \le A_{\max}^2, \tag{94}$$

where $A_{\max}$ is the maximum allowable amplitude. Note that $p(T, \omega)$ depends on the control $\alpha = u + iv$ as defined in (88). When $v = 0$, this optimal control problem, following some algebraic manipulations, can be reduced to a convex optimization problem of the form

$$\begin{aligned} \min_{u} \quad & \int_0^T \left[\int_0^T \frac{\sin[\beta(\tau - \sigma)]}{\tau - \sigma} u(\tau) u(\sigma) d\sigma + \frac{2\sin(\beta\tau)}{\tau} u(\tau) \right] d\tau \\ \text{s.t.} \quad & u^2(t) \le A_{\max}^2. \end{aligned} \tag{95}$$

Discretizing this convex problem leads to a finite-dimensional quadratic program

$$\min_{\mathbf{U}} \quad \mathbf{U}'\mathbf{H}\mathbf{U} + 2\mathbf{U}'\mathbf{Q} \tag{96}$$

$$\text{s.t.} \quad |u_i| \le 1, \quad i = 1, \ldots, n, \tag{97}$$

where $\mathbf{U} = (u_1, \ldots, u_n)'$, $t_1 = 0$, $t_n = T$,

$$\mathbf{H} = \begin{bmatrix} \text{sinc}(t_1 - t_1) & \text{sinc}(t_1 - t_2) & \ldots & \text{sinc}(t_1 - t_n) \\ \text{sinc}(t_2 - t_1) & \text{sinc}(t_2 - t_2) & \ldots & \text{sinc}(t_2 - t_n) \\ \vdots & \vdots & \ddots & \vdots \\ \text{sinc}(t_n - t_1) & \text{sinc}(t_n - t_2) & \ldots & \text{sinc}(t_n - t_n) \end{bmatrix}, \tag{98}$$

and $\mathbf{Q} = (\text{sinc}(t_1), \ldots, \text{sinc}(t_n))'$ in which $\text{sinc}(x) = \sin(x)/x$. The resulting quadratic program can be effectively solved, for example, with standard gradient methods or using commercial nonlinear programming solvers, and its global optima are in a bang-bang form, which has switching characteristics between the positive and negative maximum allowable amplitude as illustrated in Figure 3 in the manuscript.

Supplementary References

[1] R. W. Brockett. *Finite Dimensional Linear Systems.* John Wiley and Sons, Inc., 1970.

[2] J. Cavanagh, W. J. Fairbrother, A. G. Palmer, and N. J. Skelton. *Protein NMR Spectroscopy.* Academic Press, San Diego, CA, 1996.

[3] L. Ahlfors. *Complex Analysis.* McGraw Hill, 1979.

[4] E. L. Ince. *Ordinary Differential Equations.* Dover Publications, New York, 1956.

[5] J.-S Li. Ensemble control of finite-dimensional time-varying linear system. *IEEE Transactions on Automatic Control*, 56(2):345–357, 2005.

[6] C. Flammer. *Spheroidal Wave Functions.* Stanford University Press, Stanford, CA, 1957.

[7] D. B. Percival. *Spectral Analysis for Physical Applications.* Cambridge University Press, Cambridge, UK, 1993.

[8] D. Slepian and H. O. Pollak. Prolate spheroidal wave function, fourier analysis and uncertainly–i. *Bell System Tech. J.*, 40:43–64, 1961.

[9] D. Slepian and H. O. Pollak. Prolate spheroidal wave function, fourier analysis and uncertainly–v. *Bell System Tech. J.*, 57:1371–1430, 1978.

[10] A. Zlotnik and J.-S. Li. Synthesis of optimal ensemble controls for linear systems using the singular value decomposition. In *2012 American Control conference*, Montreal, June 2012.
